# Radish microgreens produced without substrate in a vertical multi-layered growing unit are rich in nutritional metabolites

**DOI:** 10.3389/fpls.2023.1236055

**Published:** 2023-09-14

**Authors:** Shimeles Tilahun, Min Woo Baek, Ki-Seok An, Han Ryul Choi, Jong Hwan Lee, Jin Sung Hong, Cheon Soon Jeong

**Affiliations:** ^1^ Agriculture and Life Science Research Institute, Kangwon National University, Chuncheon, Republic of Korea; ^2^ Department of Horticulture and Plant Sciences, Jimma University, Jimma, Ethiopia; ^3^ Interdisciplinary Program in Smart Agriculture, Kangwon National University, Chuncheon, Republic of Korea; ^4^ Department of Horticulture, Kangwon National University, Chuncheon, Republic of Korea; ^5^ Kangwon National University Eco-friendly Agricultural Product Safety Center, Chuncheon, Republic of Korea; ^6^ National Institute of Horticultural and Herbal Science, Rural Development Administration, Wanju-gun, Republic of Korea; ^7^ Department of Applied Biology, Kangwon National University, Chuncheon, Republic of Korea

**Keywords:** amino acids, antioxidant activity, glucosinolates, microgreens, radish

## Abstract

Growing microgreens on trays without substrate in a vertical multilayered growing unit offers several advantages over traditional agriculture methods. This study investigated the yield performance and nutritional quality of five selections of radish microgreens grown in sprouting trays, without a substrate using only water, in an indoor multilayer cultivation system using artificial light. Various parameters were measured, including fresh weight, dry matter, chlorophyll, minerals, amino acids, phenolics, flavonoids, anthocyanins, vitamin C, glucosinolates, and antioxidant activity with four different *in vitro* assays. After ten days, the biomass had increased by 6-10 times, and the dry matter varied from 4.75-7.65%. The highest yield was obtained from ‘Asia red’, while the lowest was from ‘Koregon red’. However, ‘Koregon red’ and ‘Asia red’ had the highest dry matter. ‘Asia red’ was found to have the highest levels of both Chls and vitamin C compared to the other cultivars, while ‘Koregon red’ exhibited the highest levels of total phenolics and flavonoids. Although variations in the levels of individual glucosinolates were observed, there were no significant differences in the total content of glucosinolates among the five cultivars. ‘Asia purple’ had the highest anthocyanin content, while ‘Asia green 2’ had the lowest. The K, Mg, and Na concentrations were significantly highest in ‘Asia green 2’, and the highest Ca was recorded in ‘Asia purple’. Overall, ‘Asia purple’ and ‘Koregon red’ were the best cultivars in terms of nutritional quality among the tested radish microgreens. These cultivars exhibited high levels of dry weight, total phenolics, flavonoids, anthocyanins, essential and total amino acids, and antioxidant activities. Moreover, the implementation of this vertical cultivation method for microgreens, which relies solely on water and seeds known for their tall shoots during the sprouting could hold promise as a sustainable approach. This method can effectively be utilized for cultivar screening and fulfilling the nutritional and functional needs of the population while minimizing the environmental impacts associated with traditional agriculture practices.

## 1 Introduction

Plant-based foods offer abundant bioactive compounds that extend well beyond essential nutrition, providing substantial health benefits (Baek et al., 2021b). Low incidences of cancer and coronary heart disease have been related to diets high in fruits and vegetables (Drewnowski and Gomez-Carneros, 2000). Considering the numerous health benefits of fruits and vegetables, the dietary guidelines for Americans, 2020-2025, recommend individuals include these food groups in half of their plates. In line with the dietary recommendation and society’s increasing interest in healthy eating, there has been an increase in demand for fresh, functional, and nutraceutical foods while still being delicious (Kyriacou et al., 2016). Microgreens, with their diverse range of colors, flavors, and textures along with their rich nutrient profile, are gaining popularity as emerging specialty crops (Poudel et al., 2023).

The practice of sprouting seeds has a long history, especially in Eastern nations, where eating seedlings is a significant part of culinary tradition (Benincasa et al., 2019), and has spread to the Western world in the past few decades (Martínez-Villaluenga et al., 2008). The germination and growth of some microgreen crops such as radish, kale, mustard, and swiss chard are fast. The time from seeding to harvest is crop dependent and may vary from one to three weeks (Di Gioia et al., 2017). Based on their age or size during harvesting and consumption, sprouted seeds can be categorized as sprouts (youngest and shortest), microgreens (intermediate, about 2 inches tall and harvested within 7-14 d), and baby greens (oldest and largest, 3-4 inch tall) (Kou et al., 2014; Poudel et al., 2023). A widely accepted definition of microgreens is “Tender immature greens produced from seeds of vegetables and herbs having fully developed cotyledons with or without the emergence of a rudimentary pair of first true leaves” (Xiao et al., 2012; Senevirathne et al., 2019). Microgreens have special qualities such as rich flavor, unique color, and concentrated bioactive compounds, which could make them a choice to garnish a wide range of main dishes or to improve the sensory qualities of salads (Kou et al., 2014; Treadwell et al., 2020; Poudel et al., 2023). As a result, they regarded as “functional foods”, which have capabilities that go beyond simply providing nutrients to health-promoting or disease-preventing properties (Xiao et al., 2012). Microgreens offer several advantages, including a short growth cycle, the possibility to use soilless cultivation, minimal space requirements, adaptability to the controlled indoor growing system, absence of phytosanitary treatments, weed control, and the need for fertilizers, and higher concentration of bioactive compounds compared to their mature counterparts (Kyriacou et al., 2016; Alloggia et al., 2023). Moreover, microgreens can play a vital role in dietary diversity and nutrient content in regions facing challenges such as water scarcity, urban areas with limited space, remote locations, and high-altitude regions characterized by seasonal variability and technical limitations (Rani et al., 2018; Singh et al., 2020).

Attaining high-quality microgreens requires careful consideration of both species and cultivar selection, as well as an understanding of how plant growth and development influence the nutritional value and phytochemical content within the same crop (Ebert, 2022; Alloggia et al., 2023). Previous studies show high variations in the profiles of GSLs, phytochemical composition, and antioxidant activities of the cruciferous vegetable family (Takaya et al., 2003), and also variations within the radish cultivars themselves (Hanlon and Barnes, 2011). Different radish cultivars showed differences in their phytochemical contents in seeds, sprouts at different ages, and mature taproots (Barillari et al., 2005; Hanlon and Barnes, 2011). Studies that compared the nutrient content of radish microgreens and their mature counterparts revealed that microgreens exhibited higher levels of nutrition than their mature parts (Xiao et al., 2012; Rani et al., 2018). Consequently, screening for radish cultivars specifically suited for microgreens production, considering both yield and nutritional quality, can offer valuable insights to meet consumers’ preferences. Notably, color stands out as a remarkable characteristic of vegetables, and radish cultivars rich in anthocyanins can draw customers’ attention with their attractive appearance including blue, purple, and red (Zhang et al., 2018). Accordingly, this study aimed to clarify the differences in the yield and nutritional quality among different colored microgreens of radish cultivars, namely ‘Asia green 1’, ‘Asia green 2’, ‘Asia red’, ‘Koregon red’, and ‘Asia purple’.

Besides, the increasing global population and the imperative to reduce the carbon footprint of food production are key drivers behind the global shift toward smart agriculture (Abaajeh et al., 2023). So, growing microgreens that contain more nutrients than their mature counterparts in vertical growing units has the potential to resolve both health and environmental challenges. Hence, the overall objective of this research was to investigate the yield performance and nutritional quality of five selections of radish microgreens grown in sprouting trays, without a substrate using only water, in an indoor multilayer cultivation system using artificial light. To compare the differences, we examined several parameters, including fresh weight, percentage of dry matter, contents of chlorophylls, minerals, amino acids, phenolics, flavonoids, anthocyanins, vitamin C, glucosinolates, and antioxidant activities by four different *in vitro* assays.

## 2 Materials and methods

### 2.1 Plant material, fresh weight, and dry matter

**Figure 1:**
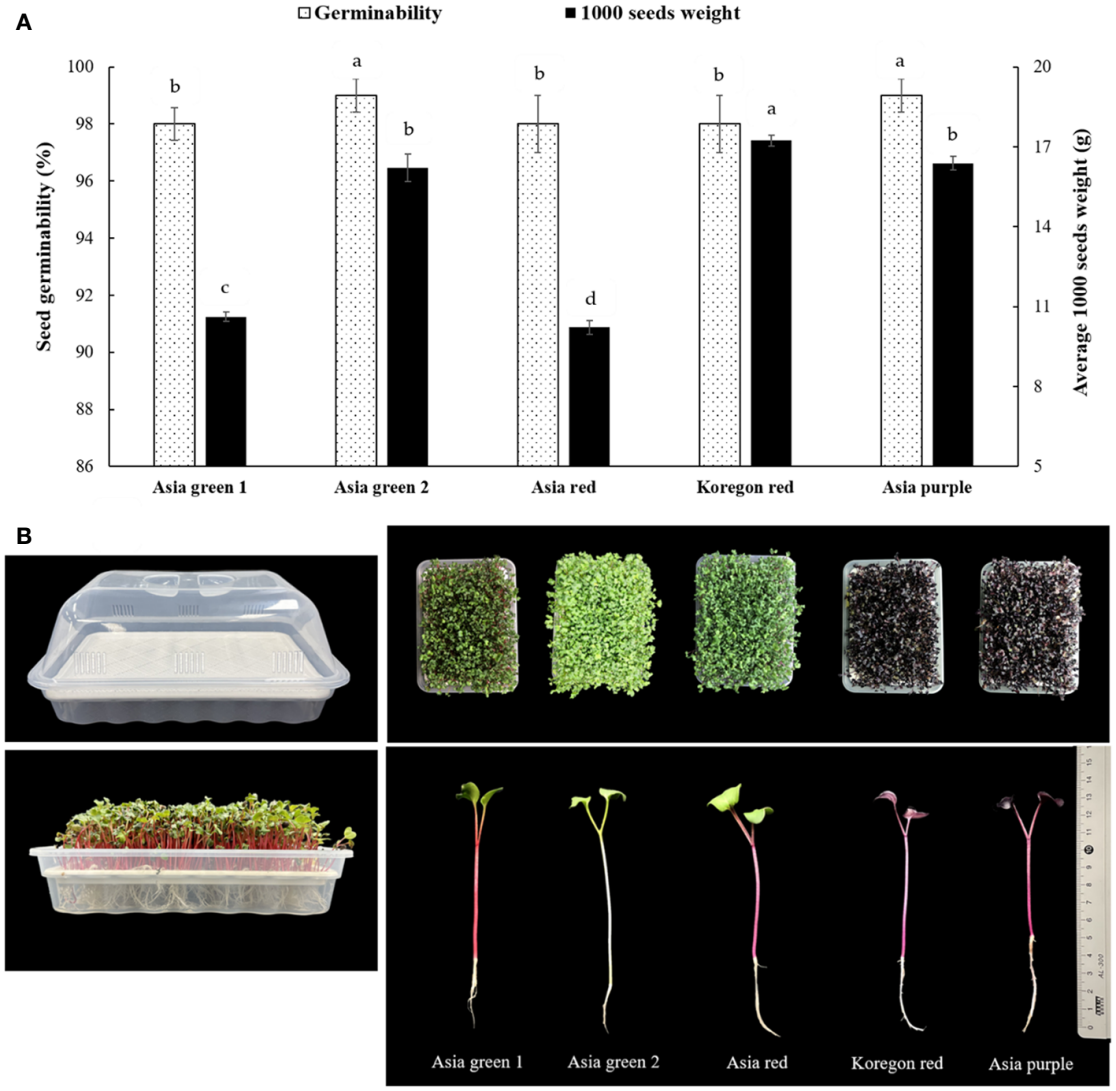
Seeds germinability and average weight of 1000 seeds **(A)**, growing trays and harvesting stages **(B)** of five radish cultivars for microgreens. Values for germinability and weight of 1000 seeds are replicates ± standard error. Significant differences at p< 0.001 were observed among cultivars in both germinability and average 1000 seeds weight. Different letters on the bars indicate significant difference between cultivars at α = 0.05 with Duncan’s mean separation procedure.

The seeds of five radish cultivars having different shoot colorations, namely ‘Asia green 1’, ‘Asia green 2’, ‘Asia red’, ‘Koregon red’, and ‘Asia purple’ were obtained from Asia Seed Co., LTD, Seoul, Korea. These cultivars are commonly distributed in Korea for the production of sprouts. Given the tendency of radish seeds to produce tall shoots, the method technically termed ‘sprouting’ was used for growing radish microgreens. There were significant variations in both germinability and average 1000 seeds weight among the five cultivars (**Figure 1A**). The average 1000 seeds weight for ‘Asia red’, ‘Asia green 1’, ‘Asia green 2’, ‘Asia purple’, and ‘Koregon red’ were recorded as 10.22, 10.63, 16.21, 16.39, and 17.23 g, respectively. The germinability of ‘Asia green 2’ and ‘Asia purple’ cultivars were found to be 99%, while ‘Asia green 1’, ‘Asia red’, and ‘Koregon red’ cultivars exhibited a germination rate of 98%. Growing trays were bought as a set consisting of a seed tray (mesh tray), water holder (lower tray), and the cover having some holes for air circulation. The size dimensions are 30 cm in length, 20 cm in width, and 15 cm in depth (including the cover) (**Figure 1B**). The seeds of each cultivar (10 g) were prepared for three replication and soaked in tap water for 24 h to enhance germination. Following the soaking period, the seeds were uniformly broadcasted on the surface of the seed trays, with a seeding density of 10 g per tray. After the seeds were uniformly distributed, the lower tray was filled with 2 L water carefully reaching the level of the mesh tray that held the seeds. This arrangement allows the seeds to rest on top of a water holder tray for fast germination and to access a continuous supply of water when the roots start to grow down. In the current study, the seeds were grown on a soilless tray having meshes to suspend the roots in the underlying pure fresh water that has been changing once per day until harvesting to avoid dirty water.

The growing trays were arranged in a vertical multilayered growing room at Kangwon National University (37°52’ N, 127°44’ E; the Republic of Korea) using a completely randomized design with three replications. The growing room consists of several growing units arranged with a spacing of 0.5 m between each unit and the wall. Each growing unit is composed 1.0 x 0.5 m shelf with four latyers, capable of accommodating six growing trays per layer. For our study, a total of 15 growing trays were used, with three trays allocated for each cultivar. These trays were arranged on three layers, with one tray from each cultivar on each layer to ensure replication. Initially, the trays were kept in a dark condition for a period of 2 days. Following the initial 2-day period, the sprouts were exposed to light intensity of 2516 lm (TL-D 32W RS 865, Philips Lighting Industry Co. Ltd, Amsterdam, Netherlands). The light cycle consisted of a 16 h light period (600 μmol m^-2^s^-1^ photosynthetic flux density (PPFD) and 6500K cool daylight spectrum) and an 8 h dark period. The temperature was maintained at 20 ± 2°C throughout the entire growth period until the microgreens reached the stage of fully developed cotyledons, just before the emergence of true leaves (**Figure 1B**).

All cultivars were harvested on the 10th day after sowing, reaching the designated harvesting stage (**Figure 1B**). Harvesting of the microgreens was done by cutting the seedlings above the mesh of the seed tray and the harvested microgreens were weighed to determine the fresh weight per tray. Then, samples were categorized and dried in a forced drought oven at 65°C to determine the dry matter. Three replicates of the remaining samples were freeze-dried with a vacuum freeze drier (FDT-8650, Operon, Korea) for analysis and the dried samples were then ground to powder. The powdered microgreen samples were sieved with 40-µm mesh, packed in LDPE pouches, and then stored at -20°C until extraction for analysis.

### 2.2 Mineral contents

Samples (1 g each) of freeze-dried microgreens of the tested radish cultivars were submitted to the National Instrumentation Center for Environmental Management (NICEM) Laboratory. Four minerals (K, Ca, Mg, and Na) were tested following microwave assisted acid digestion standard method 3051 (EPA, 2007) using inductively coupled plasma optical emission spectroscopy (5800 ICP-OES, Agilent, U.S.A).

### 2.3 Measurement of amino acids

Three samples of freeze-dried microgreens of each radish cultivar were subjected to an extraction process using 75% ethanol, ultrasonic extraction for one hour, and room temperature extraction for 24 hours. After filtering the extract with a 0.2 µL filter, an analysis of amino acid content was conducted by Dionex Ultimate 3000 HPLC using the method described by (Henderson et al., 2000).

### 2.4 Secondary metabolites

#### 2.4.1 Extraction and quantification of chlorophylls and anthocyanins content

Dimethyl sulfoxide (DMSO) chlorophyll extraction procedure was used as described by (Baek et al., 2021b). One hundred milligrams of freeze-dried radish microgreens samples were placed in a vial containing 7 mL DMSO and extraction was made by incubating at 65°C for 30 min. The absorbance readings were measured at 645 nm and 663 nm using a microplate reader (SpectraMax ABS Plus, Molecular Devices, Sunnyvale, CA, USA) against a DMSO blank. Subsequently, the total Chls, Chl a, and Chl b were determined using the equations developed by Arnon (1949) as follows:


$$\text{a}.\text{ Chl a }\left(\text{mg }{\text{g}}^{-1}\text{microgreen fres weight}\right)\;=\;\left[\left(12.7*A663\right)\;‐\;\left(2.69*A645\right)\right]\;*\;\left(\text{V}/1000*\text{W}\right)$$

$$\text{b. Chl b }\left(\text{mg }{\text{g}}^{-1}\text{microgreen fresh weight}\right)\;=\;\left[\left(22.9*\;A645\right)\;‐\;\left(4.68*\;A663\right)\right]\;*\;\left(\text{V}/1000*\text{W}\right)$$

c. Total Chls = Chl a + Chl b

V= volume of solvent W= fresh weight of the extracted tissue

Total anthocyanin (cyanidin-3-glucoside equivalents) content was analyzed using the pH differential method (Zhu et al., 2018). Freeze-dried powder of radish sprouts (0.25 g) was mixed with 5 mL of methanol containing 0.1% HCl, and then subjected to ultrasonic treatment three times for 10 minutes, the extract was centrifuged to separate the supernatant. The supernatant (50 μL) was obtained using a 0.45 μm membrane filter (PTFE, 13 mm, Whatman), and 25 mM potassium chloride buffer (pH 1.0) and 400 mM sodium acetate buffer (pH 4.5) were mixed in 950 μL, respectively, and then developed for 15 minutes. The measurements were taken at 520 nm and 700 nm using a microplate reader (SpectraMax ABS Plus, Molecular Devices, Sunnyvale, CA, USA).


$$\text{Anthocyanin }({\text{mg g}}^{-1})\;=\;\frac{\text{A}\times \text{V}\times \text{MW}\times \text{DF}}{\text{ϵ}\times \text{m}}$$

Where A, V, MW, DF, ϵ, and m stands for the difference between the absorbance values at pH 1.0 and pH 4.5 under 520 nm and 700 nm, the total volume of extract (mL), molecular weight of cyanidin-3-glucoside (449.2 g), dilution factor, molar extinction coefficient (26,900) and sample quantity (g), respectively.

#### 2.4.2 Vitamin C content

Three freeze-dried microgreen samples (1 g) of each cultivar were mixed with 10 mL of 5% meta-phosphoric acid and homogenized for 1 min. The homogenized sample was centrifuged (14,000xg for 10 min), and the liquid layer of extracts was membrane-filtered (0.22 µm) and analyzed by HPLC as described by (Kim et al., 2011) using a ZORBAX Eclipse XDB-C18 (4.6 × 250 mm, 5 µm, Agilent, Santa Clara, CA, USA) column and detector (UV-2075, Jasco, Tokyo, Japan) at 265 nm. The mobile phase, consisting of a 1:9 ratio of methanol (MeOH) to 0.1 M KH_2_PO_4_, was injected at a volume of 20 µL and flowed at a rate of 1 mL min^-1^.

#### 2.4.3 Total phenolics and flavonoids

The total phenolics and flavonoid contents of freeze-dried radish microgreen samples were measured using the methodology implemented previously in our laboratory and described by Tilahun et al. (2022). For total phenolics analysis, ethanolic extract (1 mg mL^-1^) or standard was mixed with 1 mL of 10% Folin-Ciocalteu’s phenol reagent and 1 mL of 2% sodium carbonate solution. The absorbance at 750 nm was recorded using a microplate reader (Spectramax i3, Molecular Devices, Sunnyvale, CA, USA) after incubation of the samples at ambient temperature for 90 min in the dark. The results were expressed as milligrams of gallic acid equivalents (GAE) per 100 g of sample (mg GAE 100 g^-1^) after comparison of the measurement to the calibration curve of gallic acid. For total flavonoids analysis, ethanolic extract (1 mg mL^-1^) of the extract was mixed with 1.5 mL of ethanol, 0.1 mL of 10% aluminum nitrite solution, 0.1 mL of 1-M potassium acetate solution, and 2.8 mL of distilled water. The mixture was stirred and allowed to react for 30 min. Then, the absorbance was measured at 415 nm using a microplate reader (Spectramax i3, Molecular Devices, Sunnyvale, CA, USA). The measurements were compared to a quercetin (QE) calibration curve, and the results were expressed as milligrams of QE per 100 g of sample (mg QE 100 g^-1^).

#### 2.4.4 GSLs analysis

The extraction of desulfo-GSLs from the lyophilized 200 mg sample was performed according to Ku et al. (2014). Separation and quantification of desulfo-GSLs were performed according to previously published methods by Han et al. (2019) using Dionex UltiMate 3000 ultra-high performance liquid chromatography (UHPLC) system equipped with a column oven, pump, an auto-sampler, and a diode array detector (all Thermo Fisher Scientific, Waltham, MA, USA).

### 2.5 Antioxidant activities

Freeze-dried and ground radish microgreen samples were extracted using the methodology described by (Baek et al., 2021b), which had previously been implemented in our laboratory. The DPPH radical scavenging capacity, Trolox-equivalent antioxidant capacity (ABTS), and ferric reducing antioxidant power (FRAP) were performed in triplicate according to (Baek et al., 2021b). The reducing power assay was also performed in triplicate according to the method reported by Choi et al. (2020).

### 2.6 Experimental design and data analysis

A completely randomized design with three replicates of trays was used to study the differences between cultivars during growing. After harvesting, data were collected for the above parameters with three replicates. The normality of the data was assessed in Excel, and one-way analysis of variance (ANOVA) was performed using SAS statistical software (SAS/STAT ^®^ 9.1; SAS Institute Inc., Cary, NC, USA) at a significance level of p< 0.05. To further examine the variations between cultivars, Duncan’s multiple range test was conducted. The data normalization was done by median combined with autoscaling for principal component analysis (PCA), heat map, and correlation analysis. These analyses were performed using MetaboAnalyst 5.0 software (https://www.metaboanalyst.ca/) to visualize the differences between cultivars.

## 3 Results

### 3.1 Fresh weight, dry matter and mineral contents of radish microgreens

**Figure 2:**
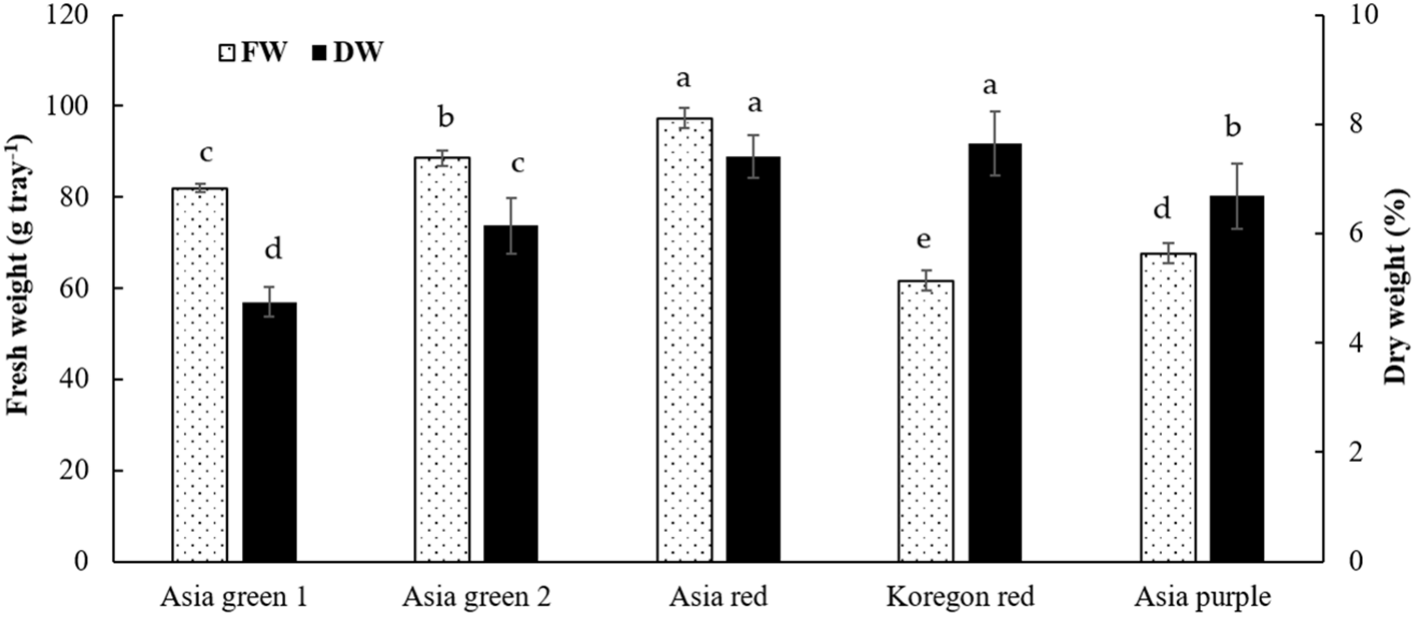
Fresh weight per tray and percentage dry weight of microgreens from five radish cultivars harvested at 10 days after sowing. Vertical bars indicate average values of three replicates ± standard error. Significant differences at p< 0.001 and 0.01 were observed among cultivars in fresh weight and percentage dry weight, respectively. Different letters on the bars indicate significant difference between cultivars at α = 0.05 with Duncan’s mean separation procedure. FW and DW, represent fresh weight and dry weight, respectively.

In this study, the average fresh yield of microgreens from 10 g seeds of each cultivar per tray were 82.0, 88.6, 97.3, 67.6, and 61.6 g for ‘Asia green 1’, ‘Asia green 2’, ‘Asia red’, ‘Asia purple’, and ‘Koregon red’, respectively, on the 10th day after sowing (**Figure 2**). A single growing unit comprises four layers on a 1.0 m x 0.5 m shelf. Each layer can hold six trays, allowing for a total of 24 trays to be arranged on one growing unit. So, from one growing unit that requires ground area of 0.5 m^2^, seed density of 240 g, and 480 L water, 1.97, 2.13, 2.33, 1.62 and 1.48 kg of biomass can be produced for ‘Asia green 1’, ‘Asia green 2’, ‘Asia red’, ‘Asia purple’, and ‘Koregon red’, respectively, within 10 days. With this vertical cultivation system of microgreens, four times space can be utilized at manageable human height than the traditional cultivation of radish on the surface. Based on the fresh weight, ‘Asia red’, ‘Asia green 2’ and ‘Asia green 1’ showed approximately 2 kg or higher fresh weight per growing unit, implying 8-10 times increments of biomass compared to the initial 10 g seed weight within 10 days. On the other hand, ‘Asia purple’, and ‘Koregon red’ showed about 1.5-1.6 kg, indicating 6 times increment of biomass within 10 days. The results showed that the cultivars that have higher average 1000 seeds weight (‘Koregon red’ and ‘Asia purple’) exhibited lower biomass compared to the cultivars that have lower average 1000 seeds weight (‘Asia red’, ‘Asia green 1’and ‘Asia green 2’) (**Figures 1A**, **2**).

In addition, significant difference was observed among the five radish cultivars in dry matter content. ‘Koregon red’ (7.65%) and ‘Asia red’ (7.41%) exhibited statistically the highest dry matter, followed by ‘Asia purple’ (6.69%) and ‘Asia green 2’ (6.14%), while the lowest (4.75%) was recorded from ‘Asia green 1’ (**Figure 2**).

**Figure 3:**
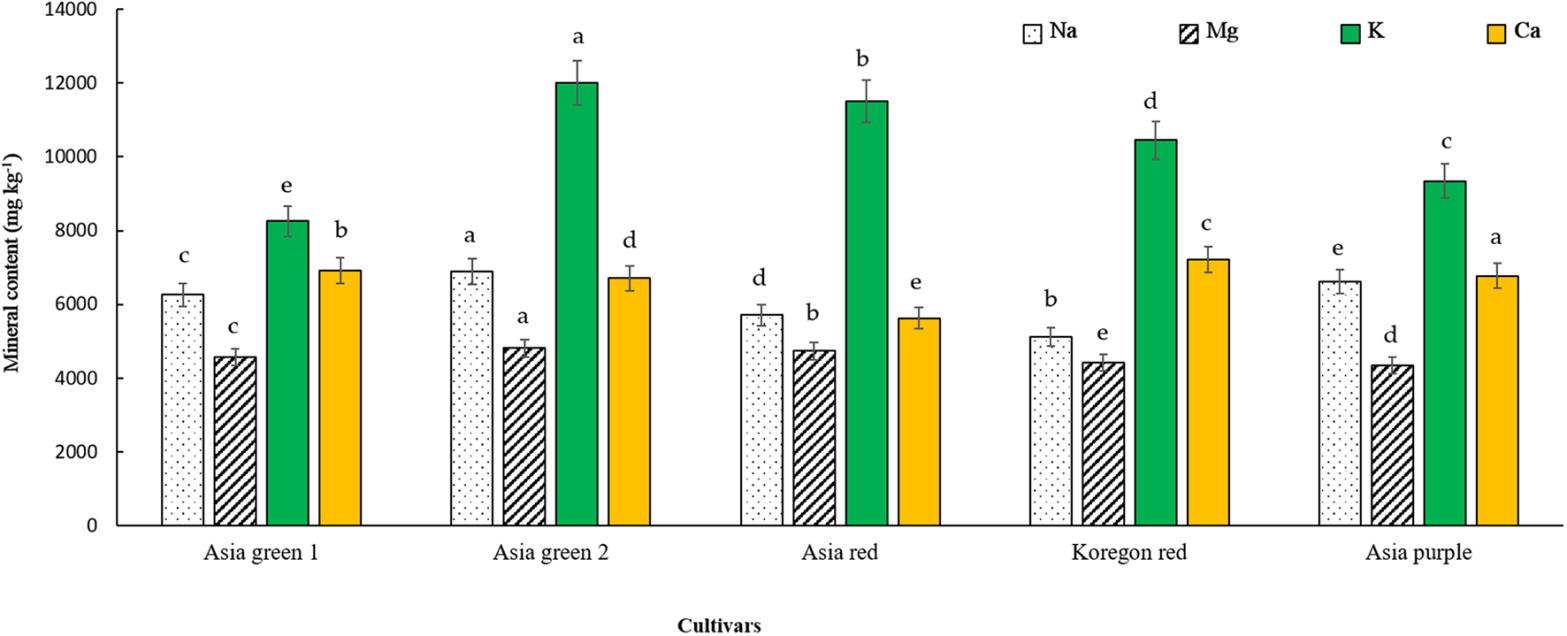
Mineral contents of microgreens from five radish cultivars harvested at 10 days after sowing. Vertical bars indicate average values of three replicates ± standard error. Significant differences at p< 0.001 were observed among cultivars in contents of Na, Mg, K, and Ca. Different letters on the bars indicate significant difference between cultivars at α = 0.05 with Duncan’s mean separation procedure.

As indicated in **Figure 3**, the concentrations of K, Mg, and Na were significantly highest in ‘Asia green 2’, and the highest Ca was recorded from ‘Asia purple’. K, Ca, Na, and Mg were ranged from 8261.67-12015.19, 5626.58-6778.41, 4427.86-4813.87, and 5111.99-6887.33 mg kg^-1^ DW, respectively. The overall results showed that K is the most abundant element in all cultivars of radish microgreens as compared to the other elements.

### 3.2 Amino acids

In this study a total of 20 amino acids were identified in the microgreens of the tested radish cultivars and the relative proportions of amino acids are significantly dependent on the cultivar (**Table 1**). Statistically higher total amino acids were recorded from ‘Asia purple’, ‘Koregon red’, and ‘Asia red’ with 86.03, 77.68, and 75.88 g kg^-1^ DW, respectively. Glutamine was the most abundant amino acid (above 39%) followed by histidine (above 16%) and asparagine (above 5%) in all the five tested cultivars. From the results of this study, total essential amino acids were higher in ‘Koregon red’ (24.57 g kg^-1^) and ‘Asia purple’ (24.46 g kg^-1^) followed by ‘Asia red’ (23.80 g kg^-1^ DW) (**Table 1**). On the other hand, the highest GABA content (0.35 g kg^-1^) was recorded from ‘Asia red’ followed by ‘Asia green 2’ (0.25 g kg^-1^) and ‘Koregon red’ (0.20 g kg^-1^ DW).

**Table 1:** Amino acids content from microgreens of five radish cultivars harvested on the tenth day after sowing.

Amino acids	Asia green 1		Asia green 2		Asia red		Koregon red		Asia purple		Significance
	mg kg^-1^ DW	%	mg kg^-1^ DW	%	mg kg^-1^ DW	%	mg kg^-1^ DW	%	mg kg^-1^ DW	%	p
Aspartic acid	612.32 ± 8.8d	0.96	799.88 ± 6.4c	1.26	918.42 ± 9.9b	1.21	946.42 ± 8.2a	1.22	896.72 ± 3.3b	1.04	***
Glutamic acid	3230.28 ± 50.3c	5.04	3133.12 ± 8.2c	4.95	3869.82 ± 41.8a	5.10	3835.47 ± 34.3a	4.94	3703.47 ± 15.6b	4.30	***
Asparagine	4187.40 ± 65d	6.53	3488.74 ± 13.5e	5.51	4913.65 ± 61.8c	6.48	6586.31 ± 56.6a	8.48	6366.60 ± 43.3b	7.40	***
Serine	1642.77 ± 27.4c	2.56	1722.68 ± 6.2b	2.72	2294.51 ± 27.1a	3.02	1375.28 ± 9.4d	1.77	1424.21 ± 7.3d	1.66	***
Glutamine	29668.69 ± 335c	46.29	27371.88 ± 107d	43.26	30858.49 ± 280b	40.67	33571.03 ± 263a	43.22	33735.82 ± 221a	39.21	***
Histidine (EAA)	11138.13 ± 252c	17.38	12637.89 ± 45.5b	19.97	10343.89 ± 143d	13.63	13529.99 ± 85.6a	17.42	13863.15 ± 108a	16.11	***
Glycine	542.12 ± 20.3b	0.85	573.38 ± 1.8ab	0.91	588.61 ± 20.4a	0.78	249.60 ± 2.7c	0.32	279.48 ± 4.3c	0.32	***
Threonine (EAA)	3188.23 ± 37b	4.97	2974.67 ± 10.8c	4.70	4008.99 ± 42.8a	5.28	2979.58 ± 15.6c	3.84	2896.77 ± 14.9c	3.37	***
Arginine	1257.29 ± 10.1d	1.96	1093.40 ± 4.2e	1.73	4878.23 ± 33.5a	6.43	3443.87 ± 27.8c	4.43	3709.47 ± 14.9b	4.31	***
Alanine	1011.30 ± 16.7c	1.58	1127.58 ± 3.2b	1.78	1383.81 ± 14.8a	1.82	968.14 ± 6.8d	1.25	1146.80 ± 7.1b	1.33	***
GABA	167.28 ± 5.1d	0.26	246.24 ± 1.6b	0.39	349.06 ± 11.9a	0.46	201.52 ± 2.9c	0.26	181.79 ± 1.6d	0.21	***
Tyrosine	545.34 ± 6.3e	0.85	843.61 ± 4.8d	1.33	1400.42 ± 16.2a	1.85	1006.71 ± 9.7c	1.30	1058.78 ± 6.7b	1.23	***
Valine (EAA)	2012.46 ± 20.2d	3.14	2159.12 ± 9c	3.41	3309.70 ± 30.6a	4.36	2690.44 ± 17.6b	3.46	2735.53 ± 13.1b	3.18	***
Methionine (EAA)	184.84 ± 1.6b	0.29	149.74 ± 1.1c	0.24	197.14 ± 4.6a	0.26	138.12 ± 0.9d	0.18	108.08 ± 1.1e	0.13	***
Tryptophane (EAA)	980.87 ± 105ab	1.53	840.03 ± 6.6b	1.33	1190.37 ± 60.8a	1.57	1148.99 ± 26.6a	1.48	798.47 ± 101b	0.93	*
Phenylalanine (EAA)	616.11 ± 1.8c	0.96	857.42 ± 3.3b	1.36	1172.63 ± 18.2a	1.55	593.33 ± 6.3cd	0.76	562.08 ± 23.6d	0.65	***
Isoleucine (EAA)	1219.32 ± 10.8c	1.90	1214.05 ± 13.1c	1.92	1825.14 ± 25.2a	2.41	1464.50 ± 10.3b	1.89	1442.26 ± 9.8b	1.68	***
Leucine (EAA)	242.19 ± 4.8e	0.38	256.95 ± 0.4d	0.41	403.04 ± 2.1b	0.53	416.56 ± 4.8a	0.54	383.90 ± 3.3c	0.45	***
Lysine (EAA)	881.87 ± 52d	1.38	985.37 ± 11.1c	1.56	1352.47 ± 27.4b	1.78	1606.65 ± 26.4a	2.07	1673.43 ± 15.7a	1.95	***
Proline	763.05 ± 85.4ab	1.19	800.91 ± 81.9ab	1.27	618.27 ± 27.1b	0.81	927.72 ± 40.6a	1.19	786.52 ± 11.9ab	0.91	*
Total EAA	20464.02 ± 450d	31.93	22075.23 ± 80.7c	34.89	23803.36 ± 234b	31.37	24568.16 ± 141a	31.63	24463.68 ± 218ab	28.43	***
Total amino acids	64091.84 ± 898b		63276.66 ± 220b		75876.63 ± 694ab		77680.22 ± 526a		86035.08 ± 875a		*

EAA represents essential amino acid. Values are presented as the mean of three replicates ± standard error. * and ***indicate significant differences at p< 0.05 and 0.001, respectively. Different letters within the same rows indicate significant difference between cultivars at α = 0.05 with Duncan’s mean separation procedure.

### 3.3 Secondary metabolites

#### 3.3.1 Chlorophylls and anthocyanins

**Figure 4:**
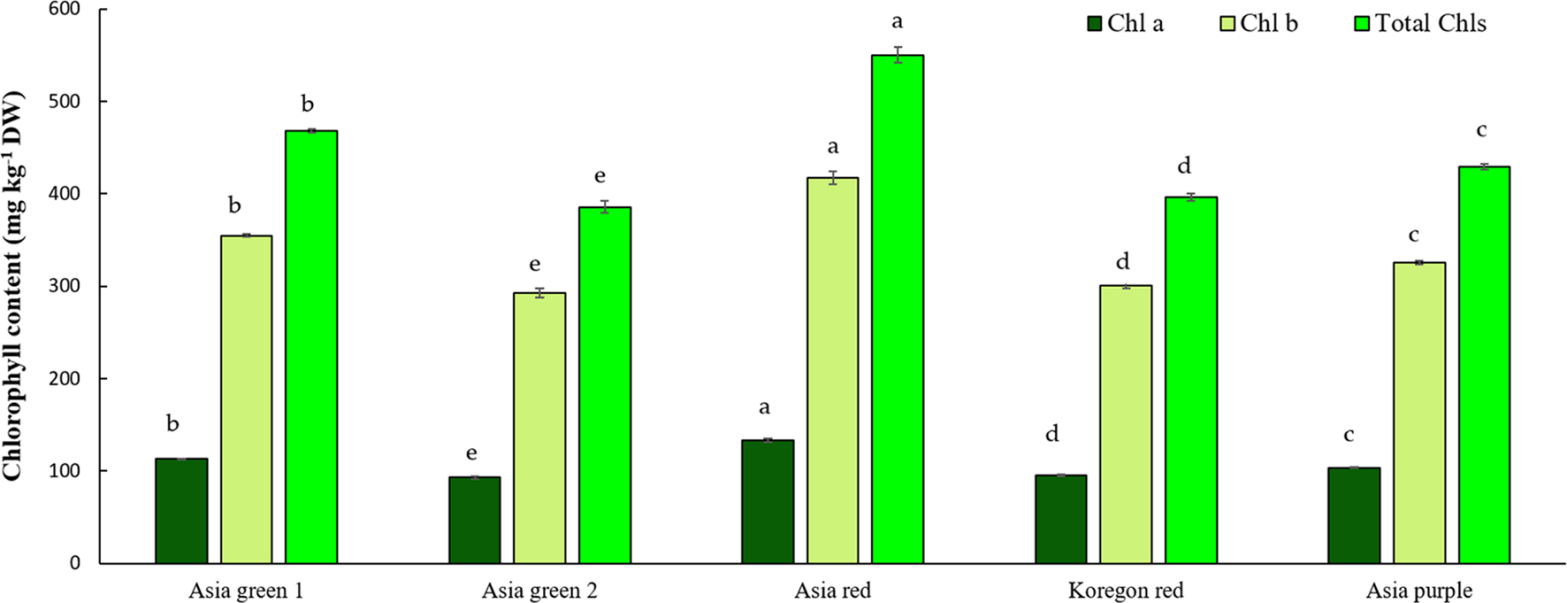
Chlorophyll contents of microgreens from five radish cultivars harvested at 10 days after sowing. Vertical bars indicate average values of three replicates ± standard error. Chl a, Chlb, and Total Chls represent chlorophyll a, b, and total chlorophylls, respectively. Significant differences at p< 0.001 were observed among cultivars in Chl a, Chlb, and Total Chls. Different letters on the bars indicate significant difference between cultivars α = 0.05 with Duncan’s mean separation procedure.

**Figure 5:**
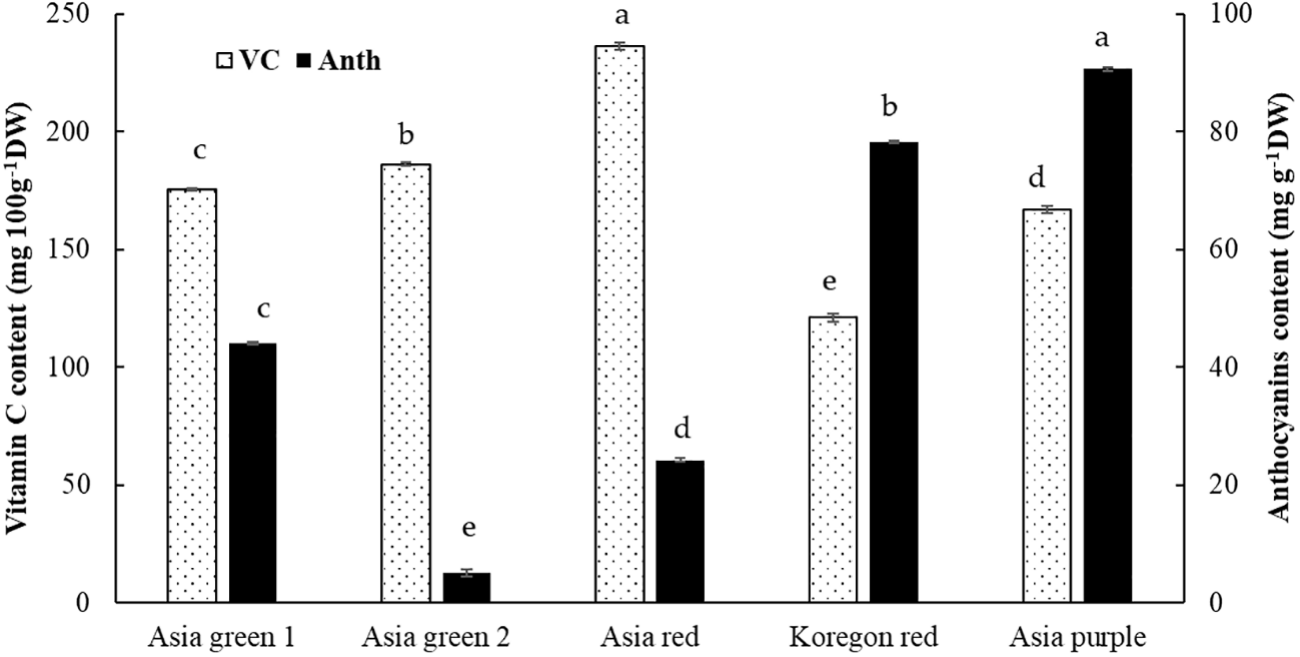
Vitamin C and anthocyanins contents of microgreens from five radish cultivars harvested at 10 days after sowing. Vertical bars indicate average values of three replicates ± standard error. Significant differences at p< 0.001 were observed among cultivars in both vitamin C and anthocyanins. Different letters on the bars indicate significant difference between cultivars α = 0.05 with Duncan’s mean separation procedure.

In this study, significant differences among radish cultivars were observed in both Chls and anthocyanins content (**Figures 4**, **5**). Total Chls and anthocyanins were ranged from 385.59-550.31 mg kg^-1^ DW and 5.02-90.65 mg g^-1^ DW, respectively. ‘Asia red’ was found to have the highest levels of Chl a, Chl b, and total Chls followed by ‘Asia green 1’. On the other hand, ‘Asia purple’ had the highest anthocyanins followed by ‘Koregon red’, while the lowest records in both Chls and anthocyanins were recorded from ‘Asia green 2’ compared to the other tested cultivars.

#### 3.3.2 Vitamin C content

In the present study, vitamin C was significantly different among the tested five cultivars of radish microgreens. From the tested cultivars, vitamin C content of ‘Asia red’ was the highest (236.27 mg 100 g^-1^ DW) followed by ‘Asia green 2’ (186.20 mg 100 g^-1^ DW), ‘Asia green 1’ (175.42 mg 100 g^-1^ DW), ‘Asia purple’ (167.04 mg 100 g^-1^ DW), and ‘Koregon red’ (121.08 mg 100 g^-1^ DW), respectively (**Figure 5**).

#### 3.3.3 Total phenolics and flavonoids content

**Figure 6:**
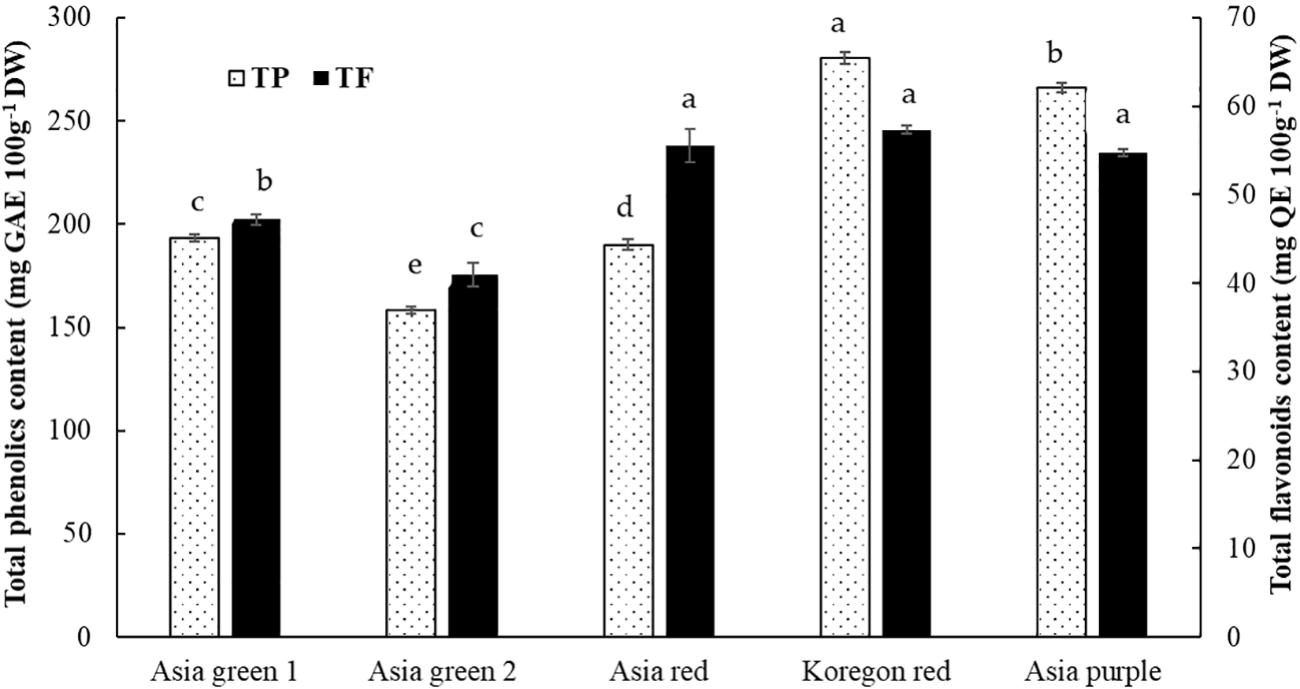
Total phenolics and flavonoids contents of microgreens from five radish cultivars harvested at 10 days after sowing. Significant differences at p< 0.001 and 0.01 were observed among cultivars in total phenolics and flavonoids, respectively. Vertical bars indicate average values of three replicates ± standard error. Different letters on the bars indicate significant difference between cultivars α = 0.05 with Duncan’s mean separation procedure.

As shown in **Figure 6**, total phenolics and flavonoids in the tested radish microgreens are dependent on cultivar. Total phenolics has significantly varied among the five studied cultivars with the highest content (280.53 mg 100 g^-1^) recorded in ‘Koregon red’ and lowest (158.15 mg 100 g^-1^) in ‘Asia green 2’. Besides, ‘Koregon red’, ‘Asia red’, and ‘Asia purple’ cultivars had statistically similar highest results of total flavonoids (57.34, 55.57, and 54.71 mg 100 g^-1^ DW, respectively), while the lowest (40.94 mg 100 g^-1^ DW) was obtained from ‘Asia green 2’.

#### 3.3.4 Glucosinolates

As presented in **Table 2**, six glucosinolates: glucoiberin, glucoraphanin, hydroxy glucobrassicin, glucoraphasatin, methoxy glucobrassicin, and neo glucobrassicin were identified in the microgreens of the five radish cultivars. Glucoraphasatin and glucoraphanin were identified predominantly in all 5 cultivars. Notably, glucoraphasatin constituted higher than 80% of the total GSLs with 82.42, 80.56, and 89.09% in ‘Asia green ‘1, ‘Asia green 2’, and ‘Asia red’, respectively. On the other hand, the content of glucoraphanin was significantly higher in ‘Asia purple’ and ‘Koregon red’ with 43.59 and 41.31%, respectively, as compared to the levels lower than 10% in the other three cultivars. The level of glucoraphasatin was also higher than other glucosinolates in ‘Asia purple’ and ‘Koregon red’ with 38.30 and 49.66%, respectively.

**Table 2:** Individual and total glucosinolates content from microgreens of five radish cultivars harvested on the tenth day after sowing.

Glucosinolates	Asia green 1		Asia green 2		Asia red		Koregon red		Asia purple		Significance
	µg g^-1^ DW	%	µg g^-1^ DW	%	µg g^-1^ DW	%	µg g^-1^ DW	%	µg g^-1^ DW	%	p
Glucoiberin	1.01 ± 0.07b	2.63	0.74 ± 0.07b	2.31	0.54 ± 0.02b	1.63	0.25 ± 0.07b	0.73	3.96 ± 1.35a	10.67	**
Glucoraphenin	3.52 ± 0.57c	9.21	3.08 ± 0.42c	9.57	1.50 ± 0.23d	4.56	14.05 ± 0.55b	41.31	16.17 ± 1.99a	43.59	***
4-Hydroxyglucobrassicin	1.10 ± 0.05b	2.87	0.73 ± 0.08c	2.26	1.04 ± 0.06b	3.15	0.78 ± 0.05bc	2.29	1.55 ± 0.32a	4.19	**
Glucoraphasatin	31.53 ± 4.37a	82.42	25.90 ± 0.39b	80.56	29.37 ± 1.88ab	89.06	16.89 ± 1.06d	49.66	14.21 ± 2.72c	38.30	***
4-Methoxygluobrassicin	0.40 ± 0.01c	1.05	0.62 ± 0.01b	1.92	0.18 ± 0.02d	0.54	1.58 ± 0.01a	4.64	0.40 ± 0.09c	1.07	***
Neoglucobrassicin	0.70 ± 0.02b	1.82	1.09 ± 0.01a	3.37	0.35 ± 0.03c	1.07	0.46 ± 0.02c	1.35	0.81 ± 0.16b	2.18	***
Total GSLs	38.26 ± 5.03a		32.15 ± 0.76a		32.98 ± 1.77a		34.01 ± 0.92a		37.09 ± 5.64a		ns

Reported values are presented as a mean of three replicates ± standard error. ns, **, and *** indicate non-significant and significant differences at p< 0.01 and 0.001, respectively. Different letters within the same rows indicate significant difference between cultivars at α = 0.05 with Duncan’s mean separation procedure.

### 3.4 Antioxidant activities

Antioxidant activities of the tested five cultivars of radish microgreens that were measured by DPPH, ABTS, FRAP and reducing power assays are shown in **Table 3**. The results showed significant differences between the cultivars, and ‘Asia purple’ showed the highest antioxidant activity followed by ‘Koregon red’ in all assays. The DPPH scavenging capacity of the radish microgreens cultivars in this study was ranging from 26.48% in ‘Asia green 2’ to 44.60 and 44.41% in ‘Koregon red’ and ‘Asia purple’, respectively, at 1mg mL^-1^ sample concentration. With the same trend, the DPPH scavenging capacity varied from 67.06% in ‘Asia green 2’ to 94.88 and 94.59% in ‘Koregon red’ and ‘Asia purple’, respectively, at 10 mg mL^-1^ sample concentration. In all the four assays, the values were concurrently higher as the sample concentration increased. Similarly, ABTS, FRAP and reducing power exhibited significantly higher values in ‘Asia purple’ and ‘Koregon red’ (**Table 3**).

**Table 3:** The DPPH (2, 2-di-phenyl-1-picrylhydrazyl) radical scavenging capacity, Trolox-equivalent antioxidant capacity (ABTS), ferric-reducing antioxidant power (FRAP), and reducing power from microgreens of five radish cultivars harvested on the tenth day after sowing.

Parameters	Sample Concentration (mg mL^-1^)	Asia green 1	Asia green 2	Asia red	Koregon red	Asia purple	Significance (p)
DPPH	1	31.63 ± 0.95b	26.48 ± 0.42c	33.61 ± 3.65b	44.60 ± 0.88a	44.41 ± 0.76a	***
	2.5	46.67 ± 0.37c	35.64 ± 0.58e	45.37 ± 0.93d	67.80 ± 0.16b	71.54 ± 0.13a	***
	5	64.78 ± 0.24c	48.01 ± 0.14e	62.73 ± 0.30d	91.91 ± 0.15b	93.10 ± 0.11a	***
	10	85.59 ± 0.05b	67.06 ± 0.33c	85.33 ± 0.11b	94.88 ± 0.38a	94.59 ± 0.07a	***
ABTS	1	12.43 ± 0.52b	10.63 ± 0.60c	12.26 ± 0.46b	16.26 ± 1.01a	16.91 ± 0.46a	***
	2.5	17.73 ± 0.78c	14.81 ± 0.92d	17.05 ± 0.89c	25.31 ± 0.53b	27.04 ± 0.33a	***
	5	25.37 ± 0.10c	19.74 ± 0.60d	24.86 ± 0.14c	39.04 ± 0.34b	41.97 ± 0.64a	***
	10	40.24 ± 0.14c	29.71 ± 0.54e	38.16 ± 0.40d	62.99 ± 0.37b	66.94 ± 0.29a	***
FRAP	1	0.06 ± 0.002d	0.06 ± 0.001e	0.07 ± 0.002c	0.13 ± 0.001b	0.14 ± 0.001a	***
	2.5	0.14 ± 0.002d	0.13 ± 0.002e	0.15 ± 0.002c	0.28 ± 0.003b	0.31 ± 0.004a	***
	5	0.28 ± 0.003c	0.23 ± 0.003d	0.28 ± 0.001c	0.54 ± 0.003b	0.58 ± 0.007a	***
	10	0.56 ± 0.004c	0.45 ± 0.004c	0.55 ± 0.007c	0.99 ± 0.074b	1.19 ± 0.114a	***
Reducing power	1	0.019 ± 0.001b	0.012 ± 0.002c	0.021 ± 0.001b	0.040 ± 0.001a	0.043 ± 0.003a	***
	2.5	0.055 ± 0.003c	0.050 ± 0.008c	0.073 ± 0.013b	0.101 ± 0.002a	0.107 ± 0.002a	***
	5	0.114 ± 0.003c	0.103 ± 0.003d	0.136 ± 0.002b	0.175 ± 0.003a	0.182 ± 0.002a	***
	10	0.208 ± 0.007d	0.200 ± 0.008d	0.241 ± 0.002c	0.308 ± 0.009b	0.330 ± 0.009a	***

Reported values are presented as a mean of three replicates ± standard error. *** indicate significant differences at p< 0.001. Different letters within the same rows indicate significant difference between cultivars at α = 0.05 with Duncan’s mean separation procedure.

### 3.5 Heat map, principal component and correlation analysis

**Figure 7:**
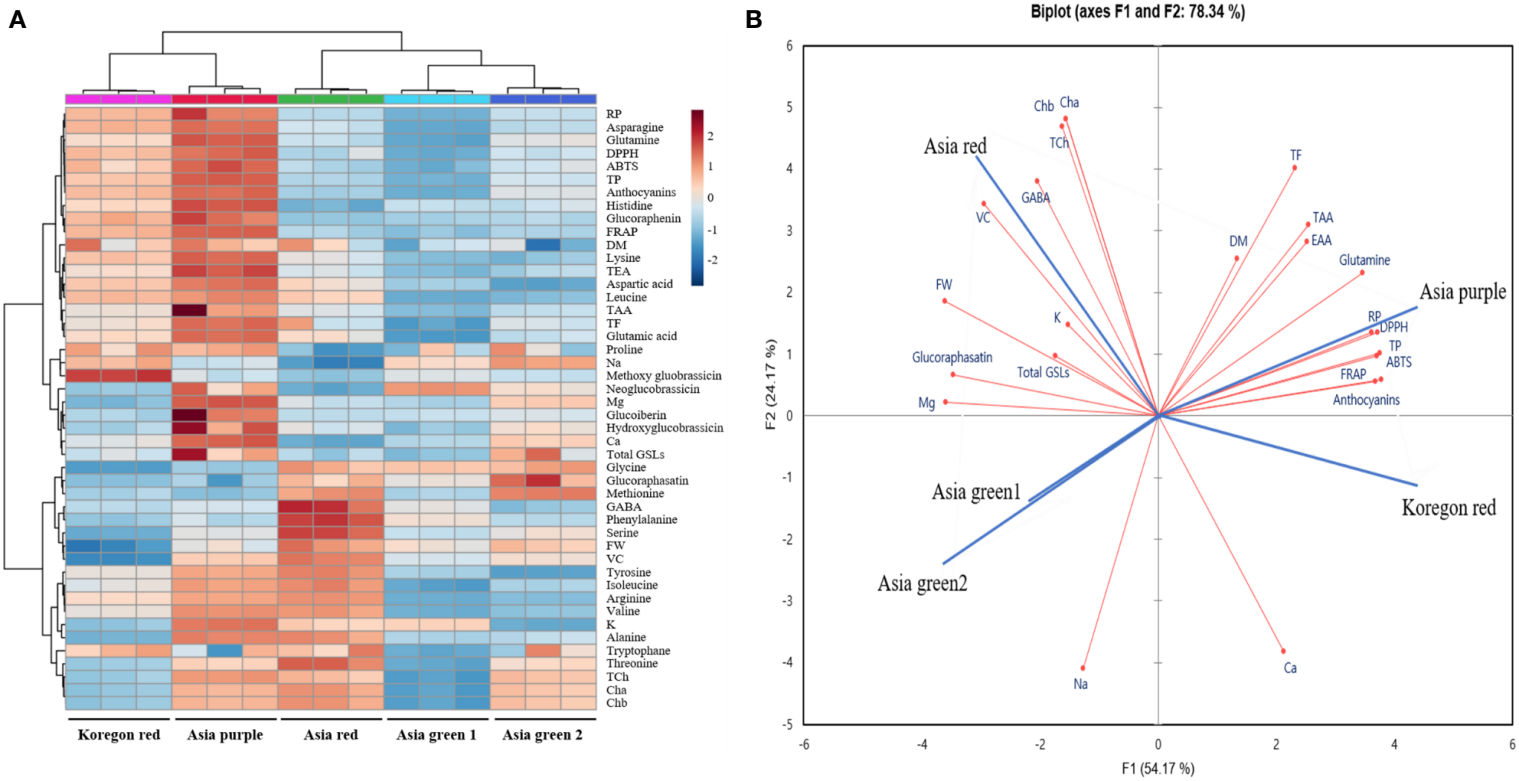
Heat map **(A)** and biplot of the principal component analysis **(B)** of the observed parameters in microgreens of five radish cultivars harvested at 10 days after sowing. • Observed parameters • Cultivars. The data normalization was done by median combined with autoscaling and these analyses were performed using MetaboAnalyst 5.0 software (https://www.metaboanalyst.ca/). FW, DM, Cha, Chb, TCh, TAA, TEA, VC, TP, TF, Total GSLs, DPPH, FRAP, ABTS, and RP represent fresh weight, dry matter, chlorophyll a, chlorophyll b, total chlorophyll, total amino acids, total essential amino acids, vitamin C, total phenolics, total flavonoids, total glucosinolates, α-diphenyl-β-picrylhydrazyl, ferric reducing antioxidant power, 2,2′-azino-bis(3-ethylbenzothiazoline-6-sulfonic acid), and reducing power, respectively.

**Figure 8:**
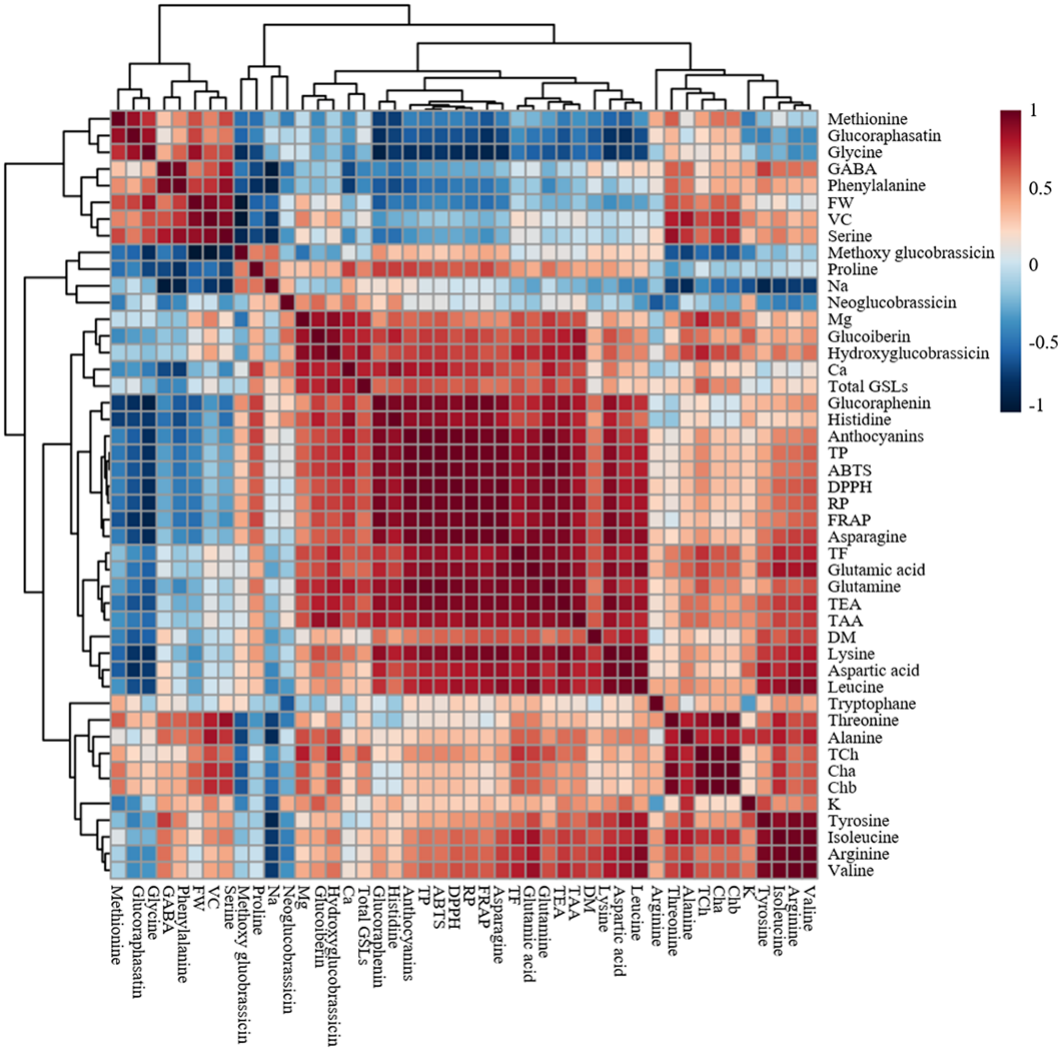
Correlation heat map of the observed parameters in microgreens of five radish cultivars harvested at 10 days after sowing. The data normalization was done by median combined with autoscaling and the analysis was performed using MetaboAnalyst 5.0 software (https://www.metaboanalyst.ca/). FW, DM, Cha, Chb, TCh, TAA, TEA, VC, TP, TF, Total GSLs, DPPH, FRAP, ABTS, and RP represent fresh weight, dry matter, chlorophyll a, chlorophyll b, total chlorophyll, total amino acids, total essential amino acids, vitamin C, total phenolics, total flavonoids, total glucosinolates, α-diphenyl-β-picrylhydrazyl, ferric reducing antioxidant power, 2,2′-azino-bis(3-ethylbenzothiazoline-6-sulfonic acid), and reducing power, respectively. Positive and negative correlations are shown as red and blue colors, respectively. The colors’ darkness’s corresponding to a higher or lower value of Pearson’s correlation. A darker color indicates stronger correlation.

Heat map, principal component analysis (PCA), and correlation analysis included all the parameters measured on the microgreens of the five radish cultivars to summarize the results presented in **Figures 2**–**6** and **Tables 1**–**3**. To identify underlying patterns or trends of the measured parameters of the radish microgreens, a heat map was generated as shown in **Figure 7A**. Higher and lower levels of each measured parameters are shown as red and blue colors, respectively and the colors’ darkness’s corresponding to the level of each parameter. A darker color indicates either the highest or the lowest. In addition, a biplot of the PCA analysis was performed to obtain a comprehensive understanding of the relationships between the cultivars, their yield, and nutritional quality (**Figure 7B**). The results revealed that the first two principal components accounted for 78.34% of the total variance, with the first principal component being the most important, explaining over 50% of the total variance. Furthermore, the correlation heatmap revealed a clear association among the collected parameters (**Figure 8**). Positive and negative correlations are shown as red and blue colors, respectively. The colors’ darkness’s corresponding to a higher or lower value of Pearson’s correlation. A darker color between the correlated parameters indicates a stronger correlation. For instance, higher positive correlations were observed between total phenolics and antioxidant activities (**Figure 8**).

## 4 Discussion

Based on our preliminary tests, it was determined that the optimal stage for harvesting the microgreens of the tested radish cultivars was on the 10th day. Harvesting earlier than the 10th day was found to have adversely affected the yield, while delayed harvesting resulted in a decline in quality, as the growth of microgreens entirely relies on the stored food reserves within the seed. After a 10-day growth period, the biomass had increased by 6-10 times, and the dry matter varied from 4.75-7.65%. With this multilayered vertical growing system, 3.94, 4.26, 4.66, 3.24, and 2.96 kg of biomass can be produced for ‘Asia green 1’, ‘Asia green 2’, ‘Asia red’, ‘Asia purple’, and ‘Koregon red’, respectively, within 10 days from two growing units that require a ground area of 1 m^2^ and a spacing of 0.5 m^2^. This implies a yield of 2.63, 2.84, 3.11, 2.16, and 1.97 kg m^-2^. Among the tested cultivars, ‘Asia red’ demonstrated the highest yield, whereas ‘Koregon red’ exhibited the lowest yield. However, in terms of dry matter content, ‘Koregon red’ and ‘Asia red’ showed the highest levels, regardless of the variations in the average 1000 seed weight. Notably, the fresh weight was inversely related to the average 1000 seed weight, as observed in ‘Koregon red’ and ‘Asia purple’, which could be attributed to the seed density. The stored food reserves within the seed are enough to attain the cotyledon stage for most vegetable microgreens (Rani et al., 2018; Treadwell et al., 2020). In agreement with our findings, Singh et al. (2020) reported yields of 95.56 and 94.89 g of fresh weight per tray from 9.05 and 10.73 g of seeds in white and pink radish cultivars, respectively, grown on a mixture of coco peat, vermiculite, and perlite in a 5:2:1 ratio and harvested on the 10th day after sowing. In addition to the yield in terms of fresh weight, effective retention of dry matter and nutritional content is crucial for choosing microgreens as alternative vegetables. The results of dry matter percentage in this study corroborated the findings reported by Wojdyło et al. (2020). According to Wojdyło et al. (2020), the high water content in ‘Asia green 1’ could be attributed to its advanced vegetative growth stage, ability to store water effectively, and adaptations to minimize water loss. Based on the findings of this study, it may be advisable to consider seed density tray^-1^ rather than seed weight tray^-1^ when producing microgreens. This is because the size of the seed, which impacts the stored food reserves could be dependent on cultivar. Regarding mineral contents, Rani et al. (2018) also reported similar trends of mineral contents and higher concentrations of K as obtained in this study in radish microgreens during their comparison of radish microgreens to their mature counterpart.

The quality of protein in food is assessed based on the levels of nine essential amino acids (EAA), namely histidine, threonine, valine, methionine, tryptophan, phenylalanine, isoleucine, leucine, and lysine (Choi et al., 2022). Additionally, GABA received more attention due to its role as an inhibitory neurotransmitter in the central nervous system and it reduces blood pressure, induces relaxation, and improves immunity (Baek et al., 2021a; Tilahun et al., 2021). Wojdyło et al. (2020) reported wide variation in total amino acid content among selected different kinds of sprouts and microgreens. Similarly, the evaluation of different cultivars of radish microgreens in the present study revealed that the contents, relative proportions, and quality of amino acids were dependent on the cultivar. Among the tested cultivars, ‘Asia purple’, ‘Koregon red’, and ‘Asia red’ exhibited superior levels of total amino acids. Likewise, ‘Koregon red’ and ‘Asia purple’ displayed higher levels of total EAA, followed by ‘Asia red’. Among the tested cultivars, ‘Asia red’ had the highest content of GABA, followed by ‘Asia green 2’ and ‘Koregon red’. This finding suggests that by screening cultivars, it is possible to identify those that are rich in high-quality amino acids.

Vegetables contain a variety of natural bioactive substances, such as vitamins, minerals, antioxidants, and pigments (Chls and carotenoids) (Baek et al., 2021b). The function of pigments is not limited to their role in capturing light for photosynthesis in living plants; they also have the ability to impact consumers preference by conveying indications of maturity, quality and freshness (Baek et al., 2021b). In addition, Chls exhibit considerable antioxidant activity (Lanfer-Marquez et al., 2005), and reduced cardiovascular disease risk factors were reported through the consumption of dietary anthocyanins (Garcia and Blesso, 2021). Therefore, contents of Chls and anthocyanins could be used as good indicator of quality in radish microgreens. Among the tested cultivars, ‘Asia red’ exhibited the highest levels of Chl a, Chl b, and total Chls followed by ‘Asia green 1’. Conversely, ‘Asia purple’ displayed the highest level of anthocyanins followed by ‘Koregon red’. In contrast, ‘Asia green 2’ showed the lowest levels of both Chls and anthocyanins compared to the other cultivars.

Yadav et al. (2019) compared the microgreens of nine summer season vegetables, and reported the maximum ascorbic acid content (52.31 mg 100 g^-1^) from ‘Aoush’ radish microgreens grown on a mixture of coco pith, perlite, and vermiculite. Similarly, Mlinarić et al. (2023) reported vitamin C varied from about 3-7.5 mg 100 g^-1^ after comparing purple, red and green radish microgreens grown on a mixture of substrate and quartz sand at different light conditions. Notably, the vitamin C content of the five cultivars grown without media in the present study surpassed the contents reported by Mlinarić et al. (2023) and Yadav et al. (2019). Similarly, in contrast to the findings reported by Mlinarić et al. (2023) for different colored radish microgreens grown on a mixture of substrate and quartz sand under varying light conditions, in the present study the radish cultivars had higher total phenolics content. Notably, these microgreens in the current study were cultivated without substrate. Our results of the total phenolics and flavonoids from the tested microgreens of the five radish cultivars grown without media are also higher than the findings reported by Yadav et al. (2019). They compared the microgreens of nine summer season vegetables, and reported the total phenolics and flavonoids contents of 135.74 and 39.83 mg 100 g^-1^, respectively, from ‘Aoush’ radish microgreens grown on a mixture of coco pith, perlite, and vermiculite. These findings suggest cultivar dependent variation in the contents of vitamin C, total phenolics, and flavonoids in radish microgreens.

In agreement with our findings, Demir et al. (2023) reported glucoraphasatin and glucoraphanin of the aliphatic group as the major glucosinolates in radish microgreens. Among the tested cultivars, glucoraphasatin and glucoraphanin constituted over 90% of the total GSLs in ‘Asia green 1’, ‘Asia green 2’, and ‘Asia red’ with a significantly higher concentration of glucoraphasatin. Similarly, the two glucosinolates account for over 80% of the total GSLs in ‘Asia purple’ and ‘Koregon red’ with relatively equal distribution. Considering the total GSLs, there were no significant differences in the glucosinolates content, except for the distribution of each glucosinolates. The variation in distribution pattern of each glucosinolates in the five cultivars could emphasize the significant influence of cultivar selection for nutritional quality in terms of glucosinolates.

The functional properties of *Brassicaceae* family that related to their rich phytochemicals is the main reason for their consumption recommendation. In addition to cancer-protective effects of glucosinolates and isothiocyanates, studies revealed the antioxidant, anti-inflammatory, anti-diabetic, neuroprotective, and cholesterol-lowering effects of *Brassicaceae* vegetables (Alloggia et al., 2023). The two cultivars ‘Koregon red’ and ‘Asia purple’ have statistically lower vitamin C content. However, the contents of total phenolics, flavonoids, and anthocyanins were significantly higher in both ‘Asia purple’ and ‘Koregon red’, while total GSLs content of all five cultivars was statistically similar. Nevertheless, the antioxidant activities of ‘Koregon red’ were either statistically similar or follow ‘Asia purple’, suggesting that GSLs, phenolics, flavonoids, and anthocyanins may have more significant protective effect than Vitamin C. The antioxidant activity of microgreens presumed to be higher due to the cumulative or the synergetic effects of these secondary metabolites rather than the individual contribution of each component (Choi et al., 2022). Generally, as nutritional quality of microgreens is mainly dependent on antioxidant activity, ‘Asia purple’ and ‘Koregon red’ are superior cultivars to be grown as microgreens on trays without media.

According to the PCA biplot and heat map analysis, the collected parameters clearly distinguished the five tested radish cultivars. For instance, higher levels of total phenolics, anthocyanins and antioxidant activities in all the four assays were clearly observed at the left top part of the heat map for ‘Koregon red’ and ‘Asia purple’ as shown on the heat map. Similarly, ‘Asia purple’ and ‘Koregon red’ were located in the upper right and lower right quadrants of PCA plot, respectively, indicating that they had higher values for most of the measured parameters such as dry matter, total phenolics, flavonoids, anthocyanins, essential and total amino acids, and antioxidant activities. On the other hand, ‘Asia red’ had higher values for fresh weight, Chls, GSLs, vitamin C, K, and Mg compared to the other four cultivars, and was located in the upper left quadrant of the PCA plot. ‘Asia green 1’ and ‘Asia green 2’, located in the lower left quadrant, had lower values for most of the measured parameters compared to the other tested cultivars. Overall, the use of PCA provided valuable insights into the relationship between the cultivars in terms of their yield and nutritional quality. Furthermore, the correlation coefficients between total phenolics and antioxidant activities were 0.93, 0.93, 0.92, and 0.92 for DPPH, ABTS, FRAP and reducing power, respectively, indicating higher positive correlation between total phenolics and antioxidant activities in all the four assays. Similarly, anthocyanins had higher positive correlation coefficients of 0.91, 0.89, 0.90, and 0.96 with DPPH, ABTS, FRAP and reducing power assays, respectively. These results suggest that the total phenolics and anthocyanins contents are the main contributors and indicators of antioxidant activities of radish microgreens.

## 5 Conclusions

This study evaluated the yield and nutritional quality of five radish cultivars grown as microgreens using a sprouting method on trays with minimal inputs (seeds and water) in a multilayered growing unit without any substrate. We have seen a four-fold increase in the yield of radish microgreens per square meter with a vertical sprouting growing system as compared to the conventional growing system. ‘Asia red’ had the highest fresh weight while ‘Koregon red’ had the lowest. However, ‘Koregon red’ and ‘Asia red’ had the highest dry matter. Overall, ‘Asia purple’ and ‘Koregon red’ were found to be the best options for obtaining high-quality nutritional content among the radish microgreens tested, as they exhibited high levels of dry weight, total phenolics, flavonoids, anthocyanins, essential and total amino acids, and antioxidant activities with four different assays. Additionally, this method of cultivating microgreens without substrate and utilizing seeds known for their tall shoots during sprouting could serve as a sustainable model for cultivar screening and meeting the nutritional and functional needs of the population. Moreover, this method has several advantages over traditional agriculture methods, such as efficient space utilization, reduced vulnerability to extreme weather and environmental conditions, elimination of the need for pesticides and fertilizers, and the ability to grow them throughout the year due to their short growing cycle. So, a vertical multilayered growing system for microgreens production could be used as one of the solutions to address the fresh vegetables need of the continuously growing world population, because, it is unaffected by adverse weather conditions and provides reliable year-round production with better use of space and minimum water usage without environmental impacts. The findings suggest that supplementing the current growing method with additional studies involving elicitor treatments on the five cultivars could be beneficial in enhancing the quality of radish microgreens. Such treatments have the potential to stimulate the production of phytochemicals (secondary metabolites), thereby increasing the antioxidant activities and resulting in value added radish microgreens.

## Author contributions

Conceptualization and methodology, ST, MWB, and CJ; data curation, JL, MWB, and K–S.A. software and formal analysis, ST and HRC.; resources, CJ; original draft preparation, ST and MWB; review and editing, JH, supervision, JH, and CJ; project administration, MWB; funding acquisition, CJ. All authors contributed to the article and approved the submitted version.
